# Pharmacodynamics of ATI-2307 in a rabbit model of cryptococcal meningoencephalitis

**DOI:** 10.1128/aac.00818-23

**Published:** 2023-09-20

**Authors:** Charles D. Giamberardino, Jennifer L. Tenor, Dena L. Toffaletti, Julia R. Palmucci, Wiley Schell, Jane-Valeriane K. Boua, Choiselle Marius, Katharine E. Stott, Shelby L. Steele, William Hope, Don Cilla, John R. Perfect

**Affiliations:** 1 Department of Medicine, Division of Infectious Diseases, Duke University, Durham, North Carolina, USA; 2 Department of Neurosurgery, Duke University, Durham, North Carolina, USA; 3 Antimicrobial Pharmacodynamics and Therapeutics, Department of Molecular and Clinical Pharmacology, University of Liverpool, Liverpool, United Kingdom; 4 Appili Therapeutics Inc., Halifax, Nova Scotia, Canada; University Children's Hospital Münster, Münster, Germany

**Keywords:** *Cryptococcus*, fungal meningitis, ATI-2307

## Abstract

Cryptococcal meningoencephalitis (CM) is a devastating fungal disease with high morbidity and mortality. The current regimen that is standard-of-care involves a combination of three different drugs administered for up to one year. There is a critical need for new therapies due to both toxicity and inadequate fungicidal activity of the currently available antifungal drugs. ATI-2307 is a novel aryl amidine that disrupts the mitochondrial membrane potential and inhibits the respiratory chain complexes of fungi—it thus represents a new mechanism for direct antifungal action. Furthermore, ATI-2307 selectively targets fungal mitochondria via a fungal-specific transporter that is not present in mammalian cells. It has very potent *in vitro* anticryptococcal activity. In this study, the efficacy of ATI-2307 was tested in a rabbit model of CM. ATI-2307 demonstrated significant fungicidal activity at dosages between 1 and 2 mg/kg/d, and these results were superior to fluconazole and similar to amphotericin B treatment. When ATI-2307 was combined with fluconazole, the antifungal effect was greater than either therapy alone. While ATI-2307 has potent anticryptococcal activity in the subarachnoid space, its ability to reduce yeasts in the brain parenchyma was relatively less over the same study period. This new drug, with its unique mechanism of fungicidal action and ability to positively interact with an azole, has demonstrated sufficient anticryptococcal potential in this experimental setting to be further evaluated in clinical studies.

## INTRODUCTION

Cryptococcal meningoencephalitis (CM) occurs when yeast cells, most frequently emanating from a respiratory source, invade the central nervous system (CNS) and begin to proliferate (1). CM primarily occurs in individuals with profound defects of cellular immunity. Even with effective antifungal therapy in resource-rich settings, 10 week mortality related to CM is approximately 20% (2). However, in some resource-limited countries, and specifically in patients with advanced HIV/AIDS, the mortality rate can reach up to 70% (3). Although worldwide cases of CM have declined since their peak in the 1990s due to effective antiretroviral therapy, there are still an estimated 300,000 cases occurring per year with over 100,000 deaths (2). The currently recommended treatment for CM in resource-rich health care systems begins with an induction phase of intravenous amphotericin B deoxycholate or lipid formulation of amphotericin B and oral flucytosine for 1 to 2 weeks, followed by a consolidation phase of daily fluconazole, for 8 weeks, and then a maintenance phase with a lower dose of daily fluconazole, which can last for up to 1 year and maybe life long without immune reconstitution (4, 5). In two recent studies in resource-limited health care systems, there was support for using abbreviated polyene-based induction regimens in some clinical settings with underlying HIV disease (6, 7). However, current therapeutic strategies are still beset by multiple issues including toxicity, compliance, drug availability, suboptimal antifungal activity, and the emergence of resistance (8). Given these limitations, there is an urgent need for further innovation in the development of anticryptococcal therapies.

Currently licensed antifungal drugs belong to one of four classes: echinocandins, polyenes, azoles, and nucleotide analogs. The echinocandins, polyenes, and azoles all target the cell membrane or cell wall (9). ATI-2307 represents a novel class of antifungal agents, whose mechanism is limited to yeasts via two mechanisms. First, ATI-2307 is preferentially taken up into fungal cells relative to mammalian cells via a high affinity carrier mechanism regulated by Agp2, which is only present in fungal cells (10, 11). Second, once internalized, ATI-2307 selectively disrupts the yeast mitochondrial membrane potential and respiratory chain resulting in rapid yeast cell death (12). While ATI-2307 collapsed the membrane potential of mitochondria isolated from *Saccharomyces cerevisiae* at 20 µM, it had no effect on the membrane potential of rat liver mitochondria at concentrations up to 10 mM, representing a >500-fold selectivity for yeast mitochondrial respiration (13). Thus, ATI-2307 has a well-understood mechanism of action that is highly targeted to important yeast cell physiology and supports its potential as a safe and potent antifungal candidate for human use.

ATI-2307 has potent *in vitro* activity against *Cryptococcus neoformans* with modal MICs (range) of 0.004–0.008 mg/L. Previous studies in mice have shown encouraging efficacy for ATI-2307 against *C. neoformans*, *C. gattii*, *Candida albicans*, and *Aspergillus fumigatus* (14 – 17). Although these findings are encouraging, ATI-2307 has not been studied in the rabbit model of CM, which is a clinically relevant mimic of human disease. For instance, the rabbit CM model is a clinically relevant for precisely testing the ability of novel anticryptococcal agents to specifically resolve CNS infections (18 – 23).

In this study, we estimated the pharmacodynamics of ATI-2307 in the rabbit model of CM by quantifying the fungal burden of *C. neoformans* in the cerebrospinal fluid (CSF) both during and upon completion of treatment with ATI-2307, amphotericin B (AMB), and fluconazole (FLU). We found a consistent reduction in the burden of yeasts in the CSF during these antifungal treatments. We also found evidence of efficacy of ATI-2307 in reducing fungal burden in brain tissue. When combined with fluconazole, it provided impressive anticryptococcal treatment. Additionally, we found that ATI-2307 given at 2 mg/kg/d for only three doses demonstrated persistent fungicidal activity, as assessed by the rate of persistent fungal burden reduction in the CSF. We discovered that ATI-2307 accumulates in the meninges and the high concentrations in this tissue (or sub-compartment) likely accounts for the persistent anticryptococcal activity once dosing is stopped.

## RESULTS

### ATI-2307 reduces fungal burden in CSF

**Fig 1:**
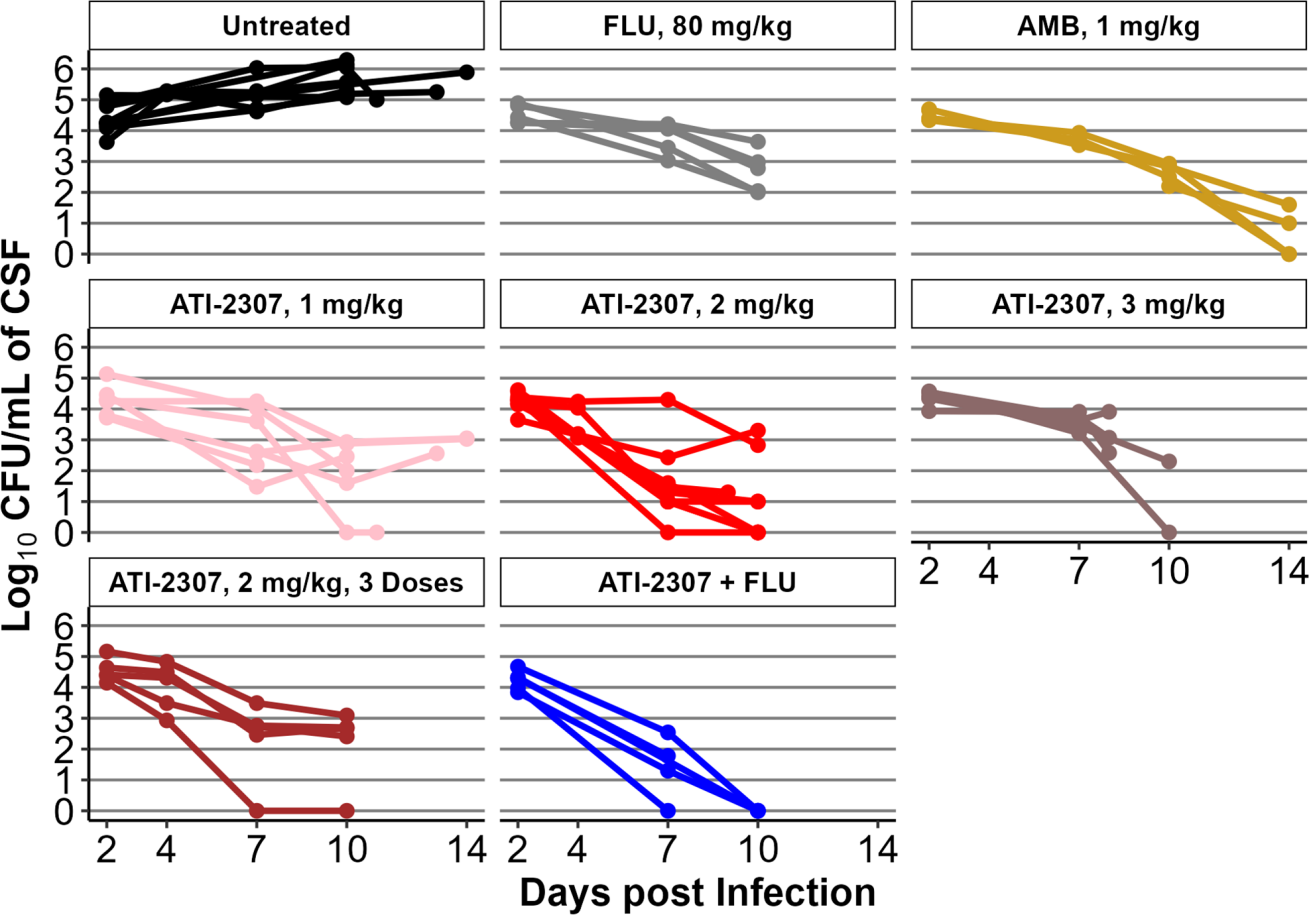
CSF Fungal Burden. Fungal burden in each individual rabbit. Untreated, *N* = 5–7; FLU, *N* = 5; AMB, *N* = 4; ATI-2307, 1 mg/kg N = 6–7; ATI-2307, 2 mg/kg, *N* = 5; ATI-2307, 3 mg/kg, N = 2 - 5; ATI-2307, 2 mg/kg, 3 Doses, N = 5; ATI-2307, 1 mg/kg + FLU, 80 mg/kg, *N* = 3–5.

To establish the efficacy of ATI-2307 in a laboratory animal model of CM, we performed three experiments in which hydrocortisone-treated rabbits were infected with *C. neoformans* intracisternally and treated with 1 mg/kg/d, 2 mg/kg/d, 3 mg/kg/d of ATI-2307 s.c., or 1 mg/kg/d ATI-2307 s.c., plus 80 mg/kg/d of FLU p.o., starting 2 days post-infection and continuing through the end of the study, days 10 or 14. In our initial experiment, we terminated the study on day 14 post infection, but due to higher weight loss in the ATI-2307-treated groups, we limited subsequent studies to 10 days (Fig. S1). The absolute fungal burden in the CSF of untreated animals increased over the 10–14 days observation period. In comparison, fungal burden in the CSF decreased in all treated groups. Relative to FLU and AMB, fungal burden in the CSF of animals treated with ATI-2307 at 1 mg/kg/d decreased at a similar rate to FLU- and AMB-treated rabbits (Fig. 1). The rabbits treated with ATI-2307 at 2 mg/kg/d initially had a much more rapid rate of fungal reduction through day 7 relative to AMB and FLU, and an average reduction in fungal burden at day 10, which was similar to FLU- and AMB-treated animals. It was noted in ATI-2307-treated groups that there were frequent small colonies observed. We suspect that these small colonies represent an impact of ATI-2307 on yeast mitochondrial function and these small colonies represent a petite cell phenotype that may represent even more yeast cell injury than viable yeast cell counts suggest (Data not shown). In the combination therapy group, the fungal burden decreased rapidly by day 7, and by day 10 for some animals (3 out of 5), fungal burdens were below the limit of detection in the CSF.

We compared fungal clearance (subtraction of log_10_ values) between day 2 and day 10 (Table 1). We limited our analyses to this range because these data were collected in all three experiments, whereas day 14 data were only collected in a single experiment. Treatment with FLU or AMB resulted in mean log_10_ CFU/mL reductions of 1.84 (SEM = 0.39) and 1.91 (SEM = 0.21), respectively; whereas treatment with 1 mg/kg/d ATI-2307 in combination with FLU resulted in a rapid loss of CSF yeast viability (Table 1). All log_10_ CFU/mL changes in treated animals were significantly different from untreated controls (*P* < 0.001, ANOVA followed by *t*-test for comparisons with Holm’s correction). However, the combination of ATI-2307 and FLU resulted in a significant mean reduction in fungal brain burden of 4.28 (SEM = 0.24) log_10_ CFU/mL and produced a significantly greater CSF yeast reduction than either FLU alone or AMB (Table 1 and Table S1).

**TABLE 1:** Change in CSF fungal burden through day 10 post infection

Group	Mean change log_10_ CFU/mL	Median change log_10_ CFU/mL	SD	*N*	SEM	95% CI upper bound	95% CI lower bound
Untreated	1.06	1.24	0.71	9	0.24	1.60	0.51
Fluconazole, 80 mg/kg	−1.84	−1.82	0.87	5	0.39	−0.76	−2.92
Amphotericin B, 1 mg/kg	−1.91	−1.86	0.41	4	0.21	−1.25	−2.56
ATI-2307, 1 mg/kg	−2.33	−2.00	1.39	5	0.62	−0.61	−4.06
ATI-2307, 2 mg/kg	−3.25	−3.86	1.52	8	0.54	−1.98	−4.52
ATI-2307, 3 mg/kg	−3.31	−3.31	1.79	2	1.27	12.77	−19.38
ATI-2307, 2 mg/kg, 3 Doses	−2.37	−2.00	1.00	5	0.45	−1.12	−3.62
ATI-2307, 1 mg/kg + FLU, 80 mg/kg	−4.28	−4.32	0.42	3	0.24	−3.24	−5.31

**Fig 2:**
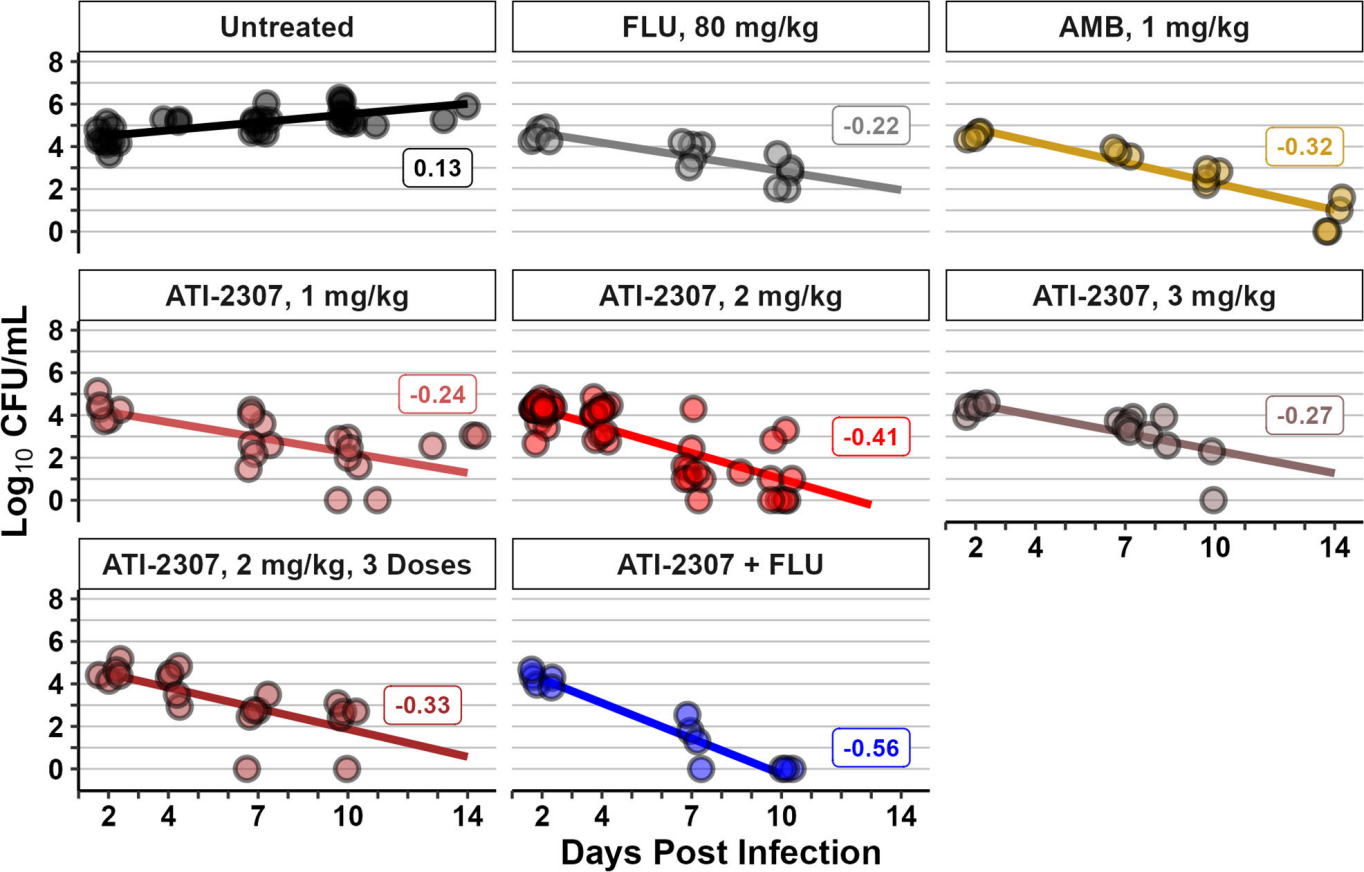
Fit of linear mixed effects model to determine EFA. A linear mixed effects model using all CSF fungal burden data. The dots are the log_10_ CFU/mL values for the fungal burden for all data (same as Fig. 1). The lines are fitted from the model using the estimated marginal means. The labels shown are the slopes of each of the lines generated from the estimated marginal means.

To specifically assess the effective fungicidal activity (EFA) of ATI-2307, we used a linear mixed effects model to test the effect of drug treatment over the course of infection using the within subject repeated measurements of fungal burden in the CSF, with the R packages lme4 and lmerTest (24, 25). The final model had the following terms: [log_10_ CFU/mL of CSF ~ Treatment + Day Post Infection + Treatment*Day Post Infection + (0 + Day Post Infection|RabbitID)], where the log_10_ CFU/mL of CSF was the dependent variable, and Treatment and Day Post Infection, along with their interaction, were the fixed effects, and a random effect term for the slope for each rabbit (summarized in Table S2). This model was compared to models without each of the fixed effects by ANOVA (data not shown). We then calculated the predicted means from the model at study days 2, 4, 7, 10, and 14 and extracted the slopes to estimate the EFA (Table 2; Fig. 2). We made pairwise comparisons of the estimated marginal means to compare different treatment regimens (Table S3). All treatment groups had *P*-values less than 0.05 when compared to the untreated rabbits. Furthermore, the Bonferroni adjusted *P*-values for the comparisons of the EFA (Table S4) in all treated groups continued to be less than 0.05 when compared to the untreated rabbits. The adjusted *P*-values of the combination treatment group of ATI-2307 (1 mg/kg/d) plus FLU (80 mg/kg/d) when contrasted with the EFAs from the linear mixed effects model of either FLU or AMB were 0.008 and 0.186, respectively.

**TABLE 2:** Slopes of estimated marginal means from linear mixed effects model

Treatment	Slope	SE	Lower limit of 95% CI	Upper limit of 95% CI
Untreated	0.13	0.04	0.04	0.21
Fluconazole, 80 mg/kg	−0.22	0.06	−0.34	−0.10
Amphotericin B, 1 mg/kg	−0.32	0.05	−0.43	−0.21
ATI-2307, 1 mg/kg	−0.24	0.05	−0.33	−0.14
ATI-2307, 2 mg/kg	−0.41	0.04	−0.49	−0.32
ATI-2307, 3 mg/kg	−0.27	0.07	−0.40	−0.14
ATI-2307, 2 mg/kg, 3 doses	−0.33	0.06	−0.44	−0.21
ATI-2307, 1 mg/kg + FLU, 80 mg/kg	−0.56	0.07	−0.69	−0.42

### ATI-2307 reduces fungal burden in brain tissue

**Fig 3:**
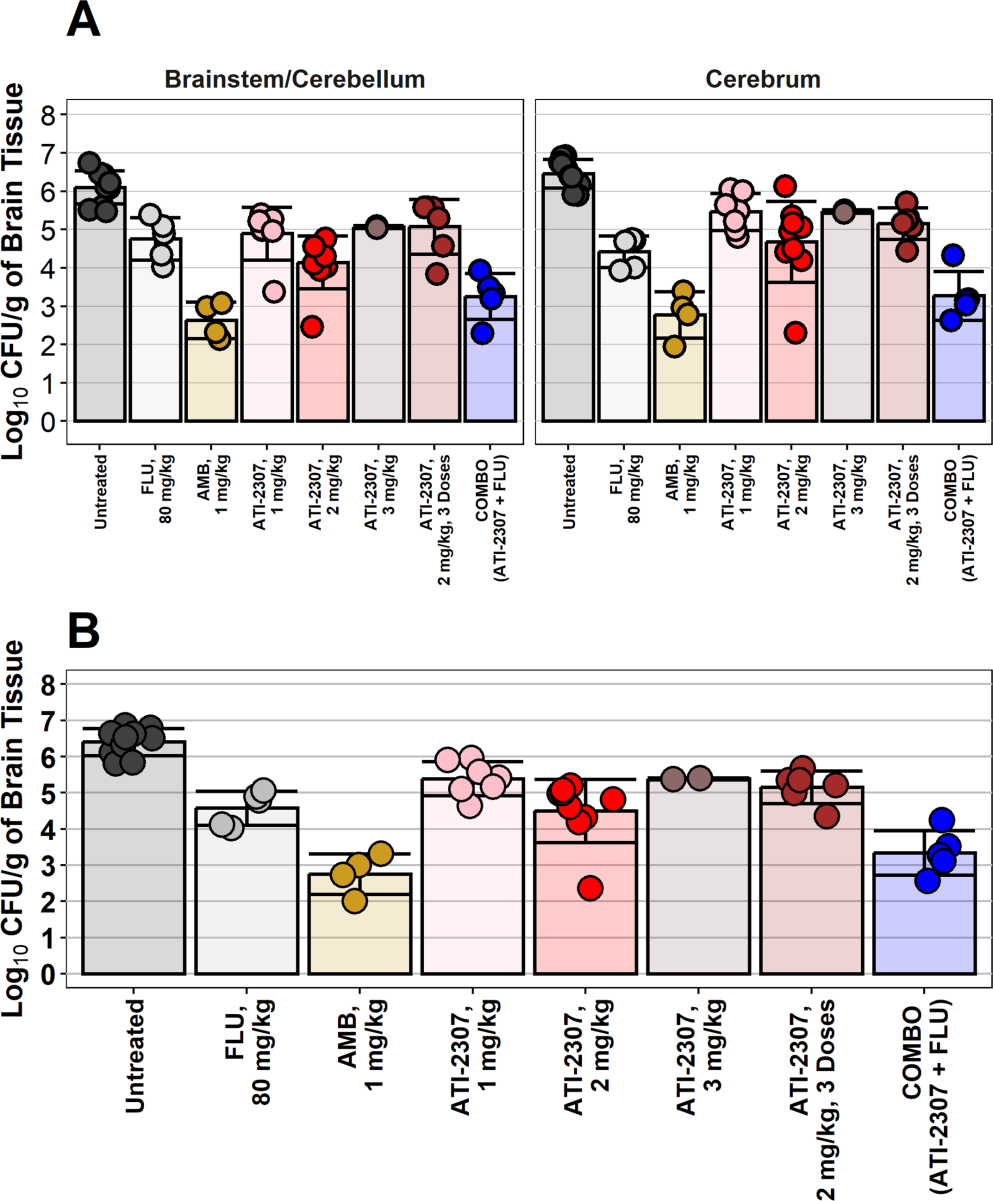
Fungal burden in brain tissue. (**A**) Fungal burden in the brain tissue separated by brain region. The fungal burden in the brain tissue was quantified. Each dot represents an individual rabbit and all data were included, regardless of the day post infection the tissue were collected. Columns represent the mean value and error bars are mean ± standard deviation. (**B**) The total brain burden was calculated by combining the burden in the brainstem/cerebellum and the cerebrum. See Table S5 for summary data including the average day post collection for the different groups. Columns represent the mean value and error bars are mean ± standard deviation.

Treatment with ATI-2307 at 2 mg/kg/d, starting at day 2 post infection and ending on day 10 post infection was equivalent to the positive control, FLU at 80 mg/kg/d over the same period in the brain. Treatment with a combination of FLU, 80 mg/kg/d and ATI-2307, 1 mg/kg/d, had superior reductions in brain CFUs relative to rabbits treated with FLU alone over the same experiment period. A shorter duration of the combination therapy (day 2 through day 10 post infection) performed similarly to AMB which was administered on day 2 through day 14 post infection. Rabbits treated with AMB received 13 doses, while rabbits treated with the combination of fluconazole and ATI-2307, 1 mg/kg/d, received only 9 doses of the combined therapy. Despite the additional four doses of AMB, the mean of the combination therapy is within 1 log_10_ CFU/g of the AMB therapy. Significantly, rabbits treated with only three doses of ATI-2307 through day 4 post infection, had greater than 1 log_10_ reductions in the brain tissue by day 10 post infection (Fig. 3; Table S5).

### ATI-2307 pharmacokinetics

In order to determine the pharmacokinetics (PK) of ATI-2307 in the rabbit CM model, PK samples were obtained on day 8 post infection (after the seventh dose of ATI-2307). Blood was collected immediately before dosing, and then 30 minutes, 1, 2, 4, 6, 8, 12, and 24 hours post dosing. The rabbits in the 3-mg/kg group had the highest absolute plasma levels. There was a linear relationship between dose and AUC_0–24_ and the maximal concentration, with increasing AUC and maximum concentrations with increasing doses from 1 to 3 mg/kg. Due to complications during the blood collections, we were unable to collect blood from all of the rabbits at all time points (Table 3).

**TABLE 3:** Pharmacokinetic profile of ATI-2307 (ng/mL) in rabbit plasma on day 8 post infection^*a*^

Group	*N*	AUC_0-24_	*C*_max_	*C*_min_	*t*_max_	*t*_last_	*t*_1/2_
ATI-2307, 1 mg/kg	6	1430 (18.7)	960 (37.7)	8.29 (28.1)	0.500 (0.500, 0.500)	24.0 (12.0, 24.0)	19.1 (5.72)
ATI-2307, 2 mg/kg	5	2350 (5.55)	1750 (10.9)	14.4 (11.2)	0.500 (0.500, 0.500)	12.0 (12.0, 12.0)	13.0 (9.81)
ATI-2307, 3 mg/kg	4	5200 (56.9)	3270 (34.0)	51.8 (42.7)	0.750 (0.500, 1.00)	13.0 (1.00, 24.0)	16.4 (5.21)
ATI-2307, 1 mg/kg + FLU, 80 mg/kg	3	1130 (30.6)	934 (18.3)	6.17 (45.4)	0.500 (0.500, 0.500)	24.0 (12.0, 24.0)	20.0 (16.0)

^*a*^ AUC, *C*_max_, and *C*_min_: geometric mean with geometric coefficient of variation in parentheses. *t*_max_, *t*_first_, *t*_last_: median with range in parentheses. *t*_1/2_: arithmetic mean with standard deviation in parentheses.

### ATI-2307 CSF and brain tissue concentrations in rabbit CM model

We first measured concentrations of ATI-2307 in the CSF on days 7 and 10 of dosing, and in the cerebrum and brainstem/cerebellum brain regions at necropsy in groups dosed with ATI-2307 alone, as well as the ATI-2307 plus FLU group (Table S6). The relationship between the dose in mg/kg of ATI-2307 and the concentration in the CSF and brain tissue was linear (concentration of ATI-2307 ~ dose of ATI-2307 mg/kg). In the CSF collected on day 7 post infection, for example, ATI-2307 concentration increases with each increasing dose (adjusted *R* ^2^ = 0.31, for the linear regression, Pearson’s *R* = 0.58 for the correlation, DF = 23, *P* = 0.002). In brain tissue (cerebrum, cerebellum/brainstem, and meninges), increases were seen between each subsequent dose level (adjusted *R* ^2^ = 0.57 for the model fit and Pearson’s *R* = 0.76 for the correlation, DF = 36, *P* = 2.91 × 10^−8^). Interestingly, ATI-2307 concentrations in the brain sections of the combination group appeared somewhat lower than the ATI-2307 alone group. We collected meninges tissue for analysis of ATI-2307 levels in one of three experiments for rabbits dosed with ATI-2307 at 2 mg/kg/d (Table S6). We observed concentrations of ATI-2307 that were far more concentrated in the meningeal tissue than in the cerebrum or in the brainstem/cerebellum (Table S6).

**Fig 4:**
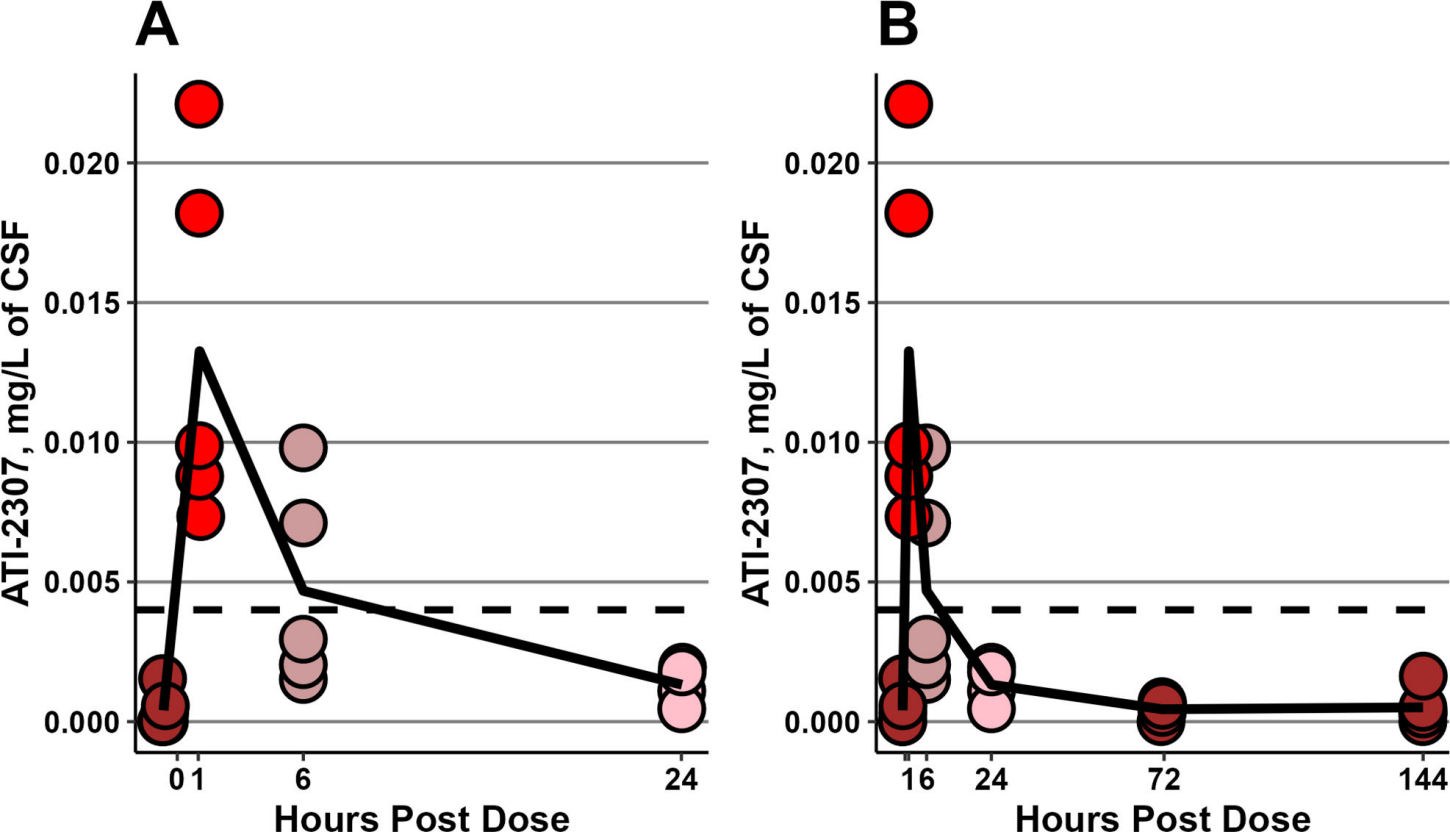
Levels of ATI-2307 in rabbit CSF. (**A**) Each dot represents data from a single rabbit. Each rabbit received doses on days 2 and 3 post infection. Trough levels for time = 0 were collected from rabbits prior to dosing on day 4 post infection. Remaining rabbits received their dose, and then a terminal CSF collection, followed by brain tissue collection. Rabbits within a time point collection group have the same color. (**B**) The same data as A with additional data from rabbits which received a single third dose, and then had CSF samples 72 and 144 hours after the dose. The black dashed line in both plots represents the MIC50 of ATI-2307, 0.004 mg/L.

**Fig 5:**
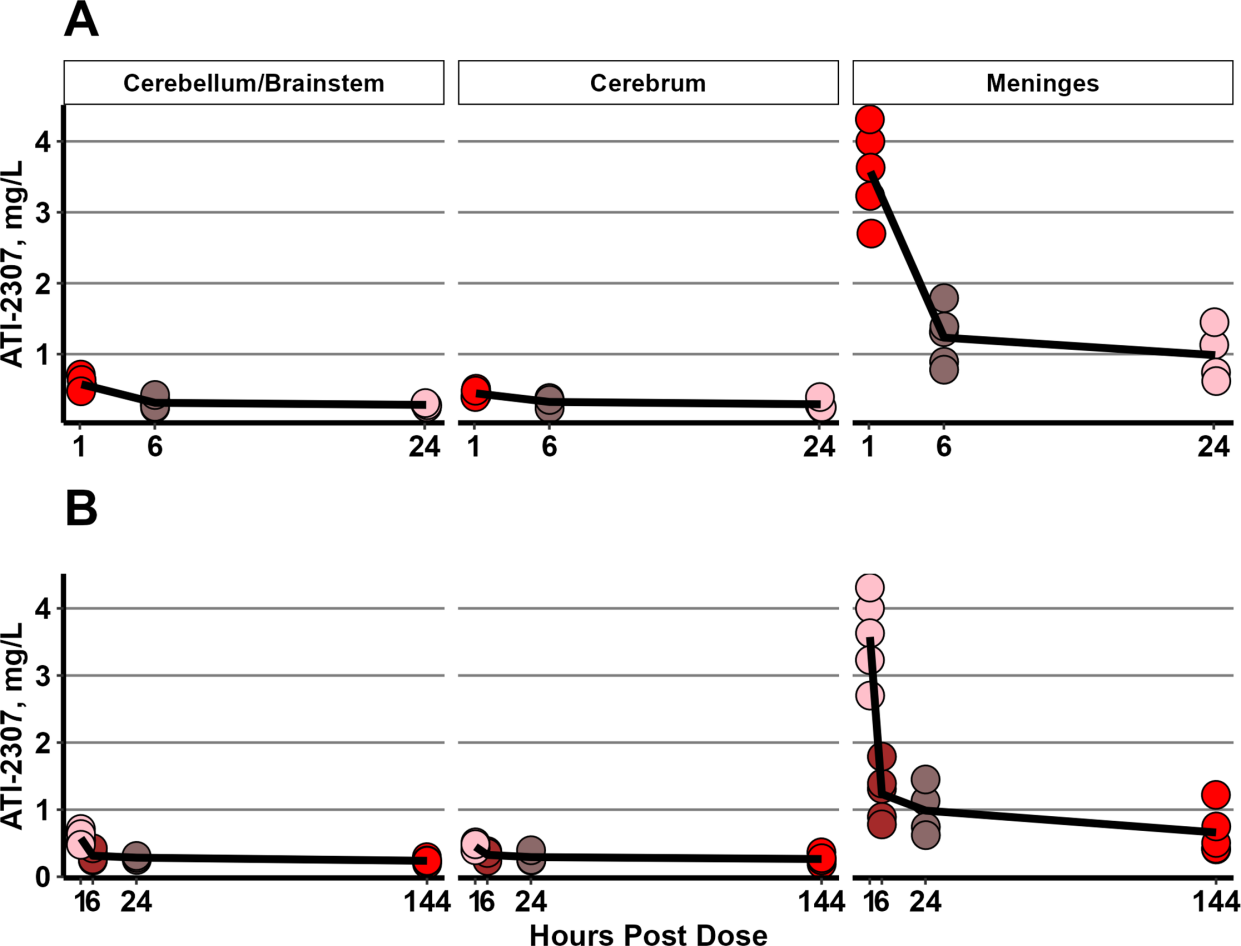
Pharmacokinetics of ATI-2307 in rabbit brain tissue compartments. (**A**) Each dot within each compartment represents data from a single rabbit (1 hour time point *n* = 4, 6 hours time point *n* = 5, 24 hours time point, *n* = 4). The lines are the mean values for the time point. The colors represent the different time cohorts, 1, 6, or 24 hours. (**B**) Data from 1 to 24 hours are replicated with additional data collected from rabbits which received three doses with tissue collected 6 days (144 hours) after the final dose.

We performed a PK experiment to determine the brain tissue and CSF kinetics of ATI-2307 given at 2 mg/kg/d and incorporating data from rabbits in the 2 mg/kg/d, 3 dose group, to determine the persistence of the drug in brain tissue with only three doses. All rabbits received doses on days 2 and 3 post infection. On day 4 post infection, *C*_min_ levels were collected from the rabbits in the ATI-2307, 2 mg/kg/d, with three doses group. These rabbits then received their third and final dose and continued on study through day 10 post infection. An additional 14 rabbits received their third 2 mg/kg dose of ATI-2307 and were then euthanized at 1, 6, or 24 hours post dose. ATI-2307 was then measured in the CSF and brain tissue (Fig. 4A and 5A). As with the terminal treatment data, the levels were highest in the meninges at all time points. We were able to extend this curve to see the persistence of the high concentrations in brain tissue after three doses up to 144 hours after the final dose (Fig. 5B) by incorporating the data from the rabbits treated with 2 mg/kg/d, three doses. From ~6 hours to ~144 hours after the third and final dose, the levels in the brain tissue remained at concentrations well above those seen in the CSF.

### Pharmacokinetic-pharmacodynamic (PK/PD) model of ATI-2307

The estimates of central tendency and dispersions for the parameters from the population PK-PD model are summarized in Table 4. The fitting of a model to the serum PK data was challenging because of the rapid distribution and relatively slower elimination phases. The fitting (especially the elimination phase) was especially sensitive to the weighting function that was used. The weighting for the PK from each rabbit was determined in ADAPT 5 using a maximum likelihood function (i.e., each rabbit had a unique set of weights that were used in the population model).

**TABLE 4:** Estimates of central tendency and dispersions for the parameters from the population PK-PD model^*a*^

Parameter (Units)	Mean	SD	CV%	Median
Ka (h^−1^)	2.561	1.161	45	3.019
SCL (L/h)	4.064	3.239	80	1.784
V (L)	6.997	9.151	131	0.532
Kcp (h^−1^)	1.684	1.235	73	0.821
Kpc (h^−1^)	1.684	2.344	139	0.030
Kpg (h^−1^)	4.473	3.014	67	6.561
Kgp (h^−1^)	4.324	0.423	10	4.113
Kgmax (log_10_CFU/mL)	0.025	0.003	14	0.027
Hg	19.388	8.599	44	19.794
C50g (mg/L)	0.032	0.019	58	0.025
Popmax (CFU/mL)	25328008.970	35325349.973	139	410581.217
Kkmax (log_10_CFU/mL)	0.301	0.111	37	0.324
Hk	23.776	3.997	17	21.962
C50k (mg/L)	0.056	0.017	30	0.065
IC (CFU/mL)	3419.797	1420.867	42	4111.459

^*a*^ See methods for definitions of each variable.

### Dose range finding of ATI-2307 in HC-treated rabbits

In these experiments, we observed more rapid weight loss and respiratory symptoms, which seemed to be dose dependent, occurring earlier and with more severity in the 2- and 3-mg/kg/d treatment groups and without changes in chemistries or blood counts (data not shown). We performed an escalating dose experiment, along with daily 5 mg/kg/d of hydrocortisone acetate, in two naïve rabbits, with 1 mg/kg on day 1, 3 mg/kg on day 2, 6 mg/kg on day 3, 8 mg/kg on day 4, and 10 mg/kg on day 5. Rabbits had lost some weight prior to the 10-mg/kg dose, but the weight loss was consistent with steroid treatment. There was some transient lethargy in rabbits after the 8-mg/kg dose, lasting less than 30 minutes. However, both rabbits went into respiratory arrest and died shortly after receiving a dose of 10 mg/kg, indicating that increases in ATI-2307 dosing cause acute toxicity and there is probably a limited range of dosing for rabbits.

## DISCUSSION

ATI-2307 had unequivocal efficacy in the rabbit treatment model for CM. The rabbit CM model is a robust pre-clinical setting for testing antifungal drug efficacy before human studies and has previously shown the efficacy of isavuconazonium sulfate, AMB, liposomal amphotericin B, fluconazole, itraconazole, APX2039, and posaconazole in this model (18 – 23, 26). The model has shown the fungistatic nature of fluconazole treatment (21), the fungicidal activity of amphotericin B despite very low CSF levels of the polyene (21), the positive CSF interaction of a polyene and azole in the CSF (22), and the ability to utilize intermittent/short doses of liposomal amphotericin B with same outcome as daily dosing (21). The ATI-2307 doses studied here compared favorably with fluconazole, and had a larger anti-fungal effect when the two were given in combination. In fact, rapid CSF sterilization occurred periodically in rabbits treated with the combination. Furthermore, the magnitude of the reduction of log_10_ CFU/mL for monotherapy with ATI-2307 was very similar to AMB in the model but less than the effect seen previously with liposomal amphotericin B (21, 26). In the literature, serial reductions of the fungal burden in the CSF (the effective fungicidal activity; EFA), during treatment is the best pre-clinical indicator for prediction of efficacy of a drug for cryptococcal meningoencephalitis in humans (27 – 29).

In the CSF pharmacokinetics analyses, ATl-2307 partitions into the CSF with inflamed meninges at between 2% and 20% of a plasma level and there is some dose dependency in CSF levels with the 2 and 3 mg/kg dosing producing higher CSF levels between 10 and 20 ng/mL. This level of drug exposure in CSF compares favorably with the extraordinarily low MIC for *C. neoformans* of 4 ng/mL (14). Furthermore, there was no evidence of a pharmacokinetic interaction between ATI-2307 and fluconazole, which supports the fact that the positive azole-effect was purely a pharmacodynamic effect.

There was some possible dose-related toxicity observed with ATI-2307 in this model. It is difficult to know if this is solely due to the drug or some combination of the immunosuppression and disease. Previous studies have shown hydrocortisone acetate in a naïve rabbit can result in weight loss, hepatic steatosis, high blood sugar, and hepatic toxicity, but it is a warning to be careful of pushing the doses of ATI-2307 without careful toxicity data.

In sum, the combination of ATI-2307 and an azole appeared to put additive stress on the yeast mitochondrial target and this leads to rapid, efficient clearing of yeasts from the subarachnoid space of the immunosuppressed rabbit. It possesses fungicidal activity and has excellent localization in the meninges for short courses. It may be optimal to utilize with an azole compound as the combination protects different tissue types and may help reduce any possible drug resistance liabilities. Thus, these encouraging positive results with ATI-2307 treatment in the rabbit model support its further studies in humans for efficacy in treatment of human CM.

## MATERIALS AND METHODS

### Rabbits

All animal experiments and methods were approved by the Duke University Institutional Animal Care and Use Committee. Male New Zealand White rabbits, 2–3 kg, (Robinson Services, Winston-Salem, NC, USA) were individually housed and allowed to acclimate for a minimum of 1 week before any procedures. Rabbits were treated with hydrocortisone acetate, 5 mg/kg, intramuscularly (IM) starting one day before infection and then daily through the end of the study. Rabbits were euthanized under sedation with ketamine (40 mg/kg) and xylazine (5 mg/kg), IM, followed by rapid IV pentobarbital/phenytoin, at the experimental endpoint (day 10 or 14 post infection) or when they showed a combination of lethargy, weight loss, respiratory, and/or gastrointestinal symptoms.

### Inoculum preparation and infection

*C. neoformans*, strain H99, was streaked onto YPD agar from glycerol stocks stored at −80°C. After 3–5 days of growth, a single colony was selected and used to inoculate YPD broth. The culture was incubated for 2 days in a 30°C shaking incubator. The cells were pelleted and washed three times in phosphate-buffered saline (PBS), and the final suspension was made in PBS (Life Technologies, Waltham, MA, USA). The yeast concentration was adjusted to approximately 3.3. × 10^6^ cells/mL, and 3 mL syringes (BD, Franklin Lakes, NJ, USA) were loaded with 0.3 mL of the inoculum and then rabbits sedated with ketamine (30 mg/kg) and xylazine (3 mg/kg) were inoculated intracisternally from a prone position.

### Cerebrospinal fluid collection

CSF was collected from all animals on day 2 post infection, prior to the first dose to establish the individual baseline fungal burden, then again on day 7 post infection (after six daily doses), day 10 (after nine daily doses). For some rabbits, CSF was also collected on day 4 post infection. In the first experiment, rabbits were treated with AMB or ATI-2307 at 1 mg/kg/d or 3 mg/kg/d, and we continued the dosing through day 14 post infection. Due to more rapid weight loss in the ATI-2307-treated rabbits, we limited the second experiment to day 10 post infection, and added a group dosed with 2 mg/kg/d (see Fig. S1). The final experimental group involved rabbits dosed with 2 mg/kg/d on day 2 through day 4 post infection, three total doses, and then euthanized on day 10 post infection. To collect the CSF, the rabbits were sedated with ketamine (30 mg/kg) and xylazine (3 mg/kg), IM, and then placed in the prone position. CSF was collected from the cisterna magna using a 3-mL syringe with 25 G 5/8″ needle. The rabbits were then given yohimbine, 0.2 mg/kg, IV, and allowed to recover.

### ATI-2307 preparation and dosing

ATI-2307 was stored at room temperature, protected from light. Solutions for injection were prepared fresh each day. The powder was weighed on an analytical balance, and then transferred to a polypropylene tube (Corning, Chelmsford, MA, USA), and wrapped in aluminum foil. The ATI-2307 was a trihydrochloride pentahydrate salt; all stock solutions were calculated for 3, 6, or 9 mg/mL of free base. The drug was dissolved in 0.9% USP saline. The rabbits were weighed and the dose was calculated to the nearest 0.01 mL, and the drug was injected subcutaneously (s.c.) once per day. On days when CSF was collected, the injections occurred approximately 1 hour before collections, with the exception of day 2, which occurred after the CSF was collected and the rabbits had fully recovered from sedation. For rabbits dosed with both ATI-2307 and FLU, the ATI-2307 was given first, and the fluconazole was given approximately 45 minutes later.

### Amphotericin B and fluconazole preparation and dosing

Amphotericin B deoxycholate, 50 mg (XGen Pharmaceuticals, Horseheads, NY, USA), was used for all polyene dosing. The lyophilized powder was reconstituted with 10 mL of USP H_2_O per manufacturer’s instructions, resulting in a 5-mg/mL stock. The stock was further diluted to final concentration of 2.5 mg/mL in 5% dextrose. The solution was aliquoted into 5 or 8 mL screw-cap tubes (Sarstedt, Nümbrecht, Germany) and stored at −20°C. On the day of injection, an aliquot was removed, wrapped in aluminum foil, and thawed at room temperature. Rabbits received a dose of 1 mg/kg.

For the intravenous (IV) injections, the rabbit’s ears were treated with lidocaine/prilocaine cream approximately 20 minutes before the injection. The rabbit was weighed, and the dose was calculated to the nearest 0.1 mL. The rabbit was secured by a holder and a second team member prepped the ear veins with 70% ethanol, and the drug was injected into the ear vein once per day.

Fluconazole (Rising, East Brunswick, NJ, USA) was purchased as a 40-mg/mL oral suspension, and prepared according to the manufacturer’s instructions. For dosing, a 20 Fr red rubber whistle-tipped catheter (VetTech, UK) was used. The rabbit was weighed and the dose was calculated to the nearest 0.2 mL. The catheter was measured from the incisors to the last rib, and then the distance was marked. A 10-mL Luer (BD) syringe was used to draw up the drug and administer it through the catheter. The volume drawn was sufficient to administer a full dose and occupy the dead space in the catheter. The catheter was then attached and the air was cleared and all dead volume was filled with drug. A holder secured the rabbit, and a second researcher carefully inserted the tube past the incisors, into the esophagus, and was inserted until the length measured to the last rib. The plunger was then depressed, and the catheter was kinked to prevent any additional volume from exiting, and then quickly removed. Each gavage took approximately 1 minute.

### PK/PD blood collection

On day 8 post infection, rabbits were sedated with acepromazine, 1–2 mg/kg, SQ, and a 21-gauge catheter was placed in the ear artery. For blood samples, a small volume, 0.2–0.3 mL, was first collected and discarded to remove any residual heparin in the port, and then a 1 mL volume was collected and immediately transferred to 1.3 mL Lithium-Heparin Tubes (Sarstedt) for drug level analysis. The tubes were shaken and then placed on ice. After the blood collection, the port was flushed with approximately 0.2 mL of heparinized saline, 100 U/mL (BD). Blood was collected for a trough level, and then the rabbits were dosed with ATI-2307. Blood was then collected at 30 minutes, 1, 2, 4, 6, 8, 12, and 24 hours post dosing.

To separate the plasma, the 1.3 mL tubes were centrifuged at 5,000 *g* × 15 minutes at 4°C. The plasma was aliquoted into 2 mL screw-cap tubes (Sarstedt) and frozen at −80°C. Blood was also collected on days 7 and 10 post infection from the ear artery using a 2-mL Lithium-Heparin vacutainer (Greiner BioOne or BD) and 23 G butterfly needle, immediately before or after CSF collection. To separate the plasma, the blood was transferred to 2 mL screw-cap tubes, and then processed as above.

For terminal blood collections, blood was collected after Euthasol was administered. A 3-mL syringe connected to an 18–20 G needle was used to collect blood via cardiac puncture. The needle was removed from the syringe and the blood was transferred to a 2-mL Lithium-Heparin vacutainer tube. The tube was inverted to mix and placed on ice. The plasma was isolated as above.

### CSF processing and CFU quantification

The CSF was transferred to 2 mL screw-cap tubes and then serially diluted in PBS and 0.1 mL volumes of the dilutions were added to YPD + chloramphenicol (100 µg/mL) agar petri dishes. If there was a large amount of blood in the CSF collection as judged by a subjective visual assessment, the data were not used for analysis. For CSF collected from rabbits which were expected to have a low burden of yeast, 0.1 mL of the CSF was directly added to the petri dish. The cultures were spread using 5 mm glass beads and then incubated for 3–7 days at 30°C. The CSF collected from animals treated with ATI-2307 produced smaller colonies than anticipated, which required longer incubation times for counting. The colonies were counted and the CFU/mL were calculated as follows: CFU counted × dilution factor / 0.1 mL. The remaining CSF which was not used for dilutions was centrifuged at 5,000 *g* × 5 minutes at room temperature. The supernatant was collected and stored at −80°C, and used for subsequent LCMS analysis.

### Necropsy and tissue processing

After euthanasia with Euthasol, rabbits were taken to necropsy. The brain and approximately 1 cm of spinal cord were removed. The brain and spinal cord were placed on a clean petri dish. A clean scalpel was used to bisect the brain and cord into right and left hemispheres. These hemispheres were cut again to separate the cerebrum from the cerebellum/brainstem. The eyes were removed from the skull and the vitreous humor was collected. The right brain side tissues, both eyes and brain, were placed into 7 mL tubes containing homogenization beads (Bertin, France) and 1–3 mL of PBS. The left-side brain tissues were placed in 8 mL screw-cap tubes. The tubes were weighed both before and after the addition of tissue to obtain net tissue weights. The left side brain tissues were placed on dry ice and then stored at −80°C.

To quantify the fungal burden, the right side brain tissues were homogenized in a Precellys Tissue Homogenizer (Bertin, France) at 6,500 rpm × 30 s. The homogenate was then serially diluted, 1:10, in PBS. The dilutions were plated onto YPD + chloramphenicol (100 µg/mL) agar petri dishes, in 0.1 mL volumes. They were incubated for 3–7 days and then the CFUs were counted. The CFU/g were calculated as follows: net tissue weight, g = tube wt with tissue, g – tube weight empty, g. Total volume, mL = net tissue weight, g + volume of PBS, mL (density assumed 1 g/mL). CFU/mL = CFU counted × dilution/0.1 mL. Total CFU = CFU/mL × total volume, mL. CFU/g = total CFU/net weight, g.

### Analysis of ATI-2307 in plasma, CSF, and brain Tissue

All plasma, CSF, and brain tissue samples were analyzed by a third-party laboratory (QPS, Newark, DE, USA). The samples were shipped on dry ice for quantification of ATI-2307 by liquid chromatography mass spectrometry (LCMS). Standard curves for plasma, CSF, and brain tissue were made in naïve rabbit plasma (BioIVT, Westbury, NY, USA), artificial CSF (Harvard Apparatus, Holliston, MA, USA), and naïve rabbit brain tissue (BioIVT, Westbury, NY, USA). Standards were dissolved in 50% acetonitrile:water. For plasma, the calibration curve range was 1 to 500 ng/mL. For CSF and brain, the calibration curves were 0.2 to 100 ng/mL. Values which were above the standard curve were extrapolated. Brain tissue was homogenized in naïve rabbit plasma (8:1, volume:mass). Samples were prepared by adding 500 µL of acetonitrile:acetic acid at 100:5 (vol:vol) to 100 µL of CSF and brain tissue, and 50 µL of plasma, and then vortex-mixed for approximately 8 minutes, and then centrifuged for approximately 15 minutes at 3,000 rpm in a Sorvall Legend RT centrifuge. The supernatant was evaporated under nitrogen and then reconstituted in 150 µL of methanol:water:formic acid at 50:50:0.5 (vol:vol:vol). T-23318 was used as the internal standard. Samples were analyzed using a Nexera X2 HPLC system (Shimadzu Corp, Duisburg, Germany) and Triple Quadrupole MS (API-4000) (Applied Biosystems, Inc., Waltham, MA, USA) mass spectrometer. One microliter of sample was injected. The columns were Atlantis HILIC Silica, 3 µm, 50 mm × 2.1 mm (Waters Corp, Milford, MA, USA). The column oven temperature was 40°C. The mobile phase consisted of two parts, A: water:methanol:acetonitrile:500 mM ammonium acetate solution (with acetic acid):acetic acid at 82:5:5:8:0.05 (vol:vol:vol:vol:vol) and B: acetonitrile:methanol:water:500 mM ammonium acetate solution (with acetic acid):acetic acid at 85:5:2:8:0.05 (vol:vol:vol:vol:vol). The autoinjector temperature was 4°C. The flow rate was 400 µL/min. The retention time for ATI-2307 was approximately 2 minutes. The mass spectrometer temperature was 600°C. The collision gas was 7 psig N_2_. The curtain gas was 30 psig N_2_. The ion source gases, 1 and 2, were both 60 psig N_2_. The ion spray voltage was 5,000 V. The entrance potential was 10 V. The scan duration was 4.4 minutes.

### PK/PD modeling

The following structural model was fitted to the PK-PD data obtained from each rabbit:


(Eq. 1) $$XP(1)=-Ka^{*}X(1)$$

(Eq. 2) $$XP(2)=Ka^{*}X(1)-(Kcp+(SCL/V){)}^{*}X(2)+Kpc^{*}X(3)-Kpg^{*}X(2)+Kgp^{*}X(4)\;$$

(Eq. 3) $$XP(3)=Kcp^{*}X(2)-Kpc^{*}X(3)$$

(Eq. 4) $$XP(4)=K{pg}^{*}X(2)-K{gp}^{*}X(4)$$

(Eq. 5) $$XP(5)={\mathrm{Kgmax}}^{*}{\left(1-\left((X(2)/V{)}^{**}Hg/\left({C50g}^{**}Hg+(X(2)/V{)}^{**}Hg\right)\right)\right)}^{*}\;\;\;\;\;\;\;(1-(X(5)/\text{ popmax }){)}^{*}X(5)-{kkmax}^{*}(X(2)/V{)}^{**}\;\;\;\;\;\;\;Hk/{\left(C50k^{**}Hk+(X(2)/V{)}^{**}Hk\right)}^{*}X(5)$$

1 [Disp-formula uFD2] [Disp-formula uFD3] - 4 describe the plasma pharmacokinetics of ATI2307. Equation 1 describes the absorption of orally administered drug from the gut to the central compartment; equation 2 is the rate of change of mass in the central compartment; equations 3 and 4 represent the rate of change into a shallow and deeper peripheral compartment to enable the relatively slow elimination of drug to be modeled. Ka is the first-order absorption rate constant; Kcp (central to peripheral rate), Kpc (peripheral to central rate), Kpg and Kgp are the respective intercompartmental rate constants; SCL is the clearance from the central compartment; and V is the volume of the central compartment.

Equation 5 describes the rate of change of fungal density in the CSF with terms that describe the rate of fungal growth that is affected by plasma drug concentrations and an explicit term that describes drug-induced fungal killing. Kgmax is the maximum rate of fungal growth; C50g is the plasma concentration that causes half-maximal decrease in the rate of growth; popmax is the maximally achievable fungal density in the CSF; kkmax is the maximum rate of drug-induced fungal killing; and Hg and Hk are the respective slope functions.

There were two output equations:


(Eq. 6) Y(1)=X(2)/V

(Eq. 7) Y(2)=DLOG⁡10(X(5))

Equation (6) describes the plasma concentrations of ATI2307 and equation (7) describes the fungal density in CSF.

The weighting functions for the PK for each rabbit were estimated in ADAPT5 using the maximum likelihood estimator.

### Data analysis

Data were manually entered into MS Excel (Redmond, WA, USA), in a shared cloud-based file (Box Inc, Redwood City, CA, USA). The results from three independent experiments were combined to generate the figures. The CFU/mL were calculated as above along with the CFU/g, using MS Excel. The log_10_ values were calculated in MS Excel using the LOG10 function. For CFU values equal to 0, the result was entered as 0 in place of the LOG_10_ error result. The data were exported as CSV files and then analyzed and graphed using R (version 4.2). The following R packages were used for analysis and figure creations: tidyverse (version 1.3.1), rstatix (version 0.7.0), ggpubr (version 0.4.0), lme4 (version 1.1.27.1), emmeans (version 1.6.4), and sjPlot (version 2.8.9). The PK parameters were calculated using the R package PKNCA (version 0.10.1). CSV tables were read as R data frames. For the brain tissue fungal burden and change in log_10_ CFU/mL of CSF, a Shapiro test was performed to confirm normality of the data, then an ANOVA test with a post-hoc *t*-test was performed in R. The pairwise comparisons of the estimated marginal means of the group factors in the linear mixed effects model were performed using the emmeans package with Bonferoni’s adjustment.

## SUPPLEMENTAL MATERIAL

Supplemental Figure 1 aac.00818-23-s0001.tif Rabbit weights as a percentage of maximum body weight. Each line represents data from a single rabbit.

Supplemental Table 1 aac.00818-23-s0002.docx Results of pairwise comparisons of change in fungal burden.

Supplemental Table 2 aac.00818-23-s0003.docx Linear mixed effects model summary.

Supplemental Table 3 aac.00818-23-s0004.docx Results of pairwise comparisons of estimated marginal means.

Supplemental Table 4 aac.00818-23-s0005.docx Results of Pairwise Comparisons of EFAs.

Supplemental Table 5 aac.00818-23-s0006.docx Brain Tissue Fungal Burden and Summary Statistics.

Supplemental Table 6 aac.00818-23-s0007.docx Brain Tissue Concentrations and Collection Times.

ASM does not own the copyrights to Supplemental Material that may be linked to, or accessed through, an article. The authors have granted ASM a non-exclusive, world-wide license to publish the Supplemental Material files. Please contact the corresponding author directly for reuse.
