# Supplementary figures and images for: Pharmacodynamics of ATI-2307 in a rabbit model of cryptococcal meningoencephalitis
Source: Antimicrob Agents Chemother. 2023 Sep 20;67(10):e00818-23. doi: 10.1128/aac.00818-23 (PMC10583688; doi:10.1128/aac.00818-23)

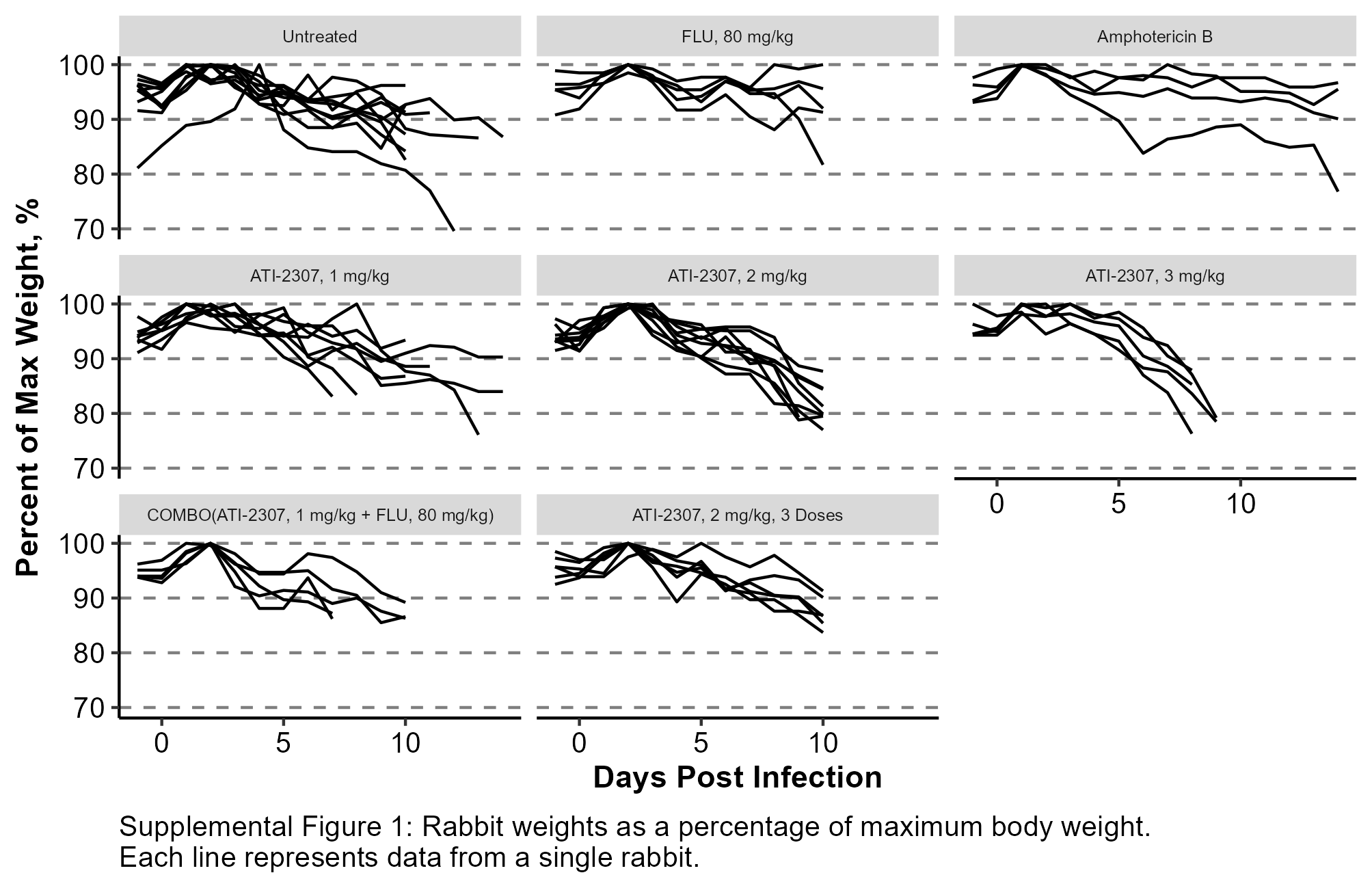

Supplement: Supplemental Figure 1 — Rabbit weights as a percentage of maximum body weight. Each line represents data from a single rabbit. [file aac.00818-23-s0001.tif]
