# Supplementary material for: Pharmacodynamics of ATI-2307 in a rabbit model of cryptococcal meningoencephalitis
Source: Antimicrob Agents Chemother. 2023 Sep 20;67(10):e00818-23. doi: 10.1128/aac.00818-23 (PMC10583688; doi:10.1128/aac.00818-23)
Supplement: Supplemental Table 1 — Results of pairwise comparisons of change in fungal burden. [file aac.00818-23-s0002.docx]

| Supplemental Table 1: Results of Change in CSF Fungal Burden T Tests with Holm’s Correction | | | | | | | |
| --- | --- | --- | --- | --- | --- | --- | --- |
| group1 | group2 | n1 | n2 | statistic | df | p | p.adj |
| ***Untreated*** | ***ATI-2307, 1 mg/kg*** | ***9*** | ***5*** | ***5.091971*** | ***5.181317*** | ***0.003*** | ***0.027*** |
| ***Untreated*** | ***ATI-2307, 2 mg/kg*** | ***9*** | ***8*** | ***7.353055*** | ***9.665594*** | ***2.94E-05*** | ***0.000382*** |
| ***Untreated*** | ***ATI-2307, 2 mg/kg, 3 Doses*** | ***9*** | ***5*** | ***6.752921*** | ***6.276654*** | ***0.000425*** | ***0.0051*** |
| FLU, 80 mg/kg | ATI-2307, 1 mg/kg | 5 | 5 | 0.672482 | 6.71969 | 0.524 | 1 |
| FLU, 80 mg/kg | ATI-2307, 2 mg/kg | 5 | 8 | 2.127433 | 10.98544 | 0.057 | 0.399 |
| FLU, 80 mg/kg | ATI-2307, 2 mg/kg, 3 Doses | 5 | 5 | 0.894132 | 7.843942 | 0.398 | 1 |
| Amphotericin B | ATI-2307, 1 mg/kg | 4 | 5 | 0.650274 | 4.849301 | 0.545 | 1 |
| Amphotericin B | ATI-2307, 2 mg/kg | 4 | 8 | 2.337649 | 8.779414 | 0.045 | 0.36 |
| Amphotericin B | ATI-2307, 2 mg/kg, 3 Doses | 4 | 5 | 0.939269 | 5.536551 | 0.387 | 1 |
| ***FLU, 80 mg/kg*** | ***ATI-2307, 1 mg/kg + FLU, 80 mg/kg*** | ***5*** | ***3*** | ***5.318284*** | ***5.911687*** | ***0.002*** | ***0.02*** |
| ***Amphotericin B*** | ***ATI-2307, 1 mg/kg + FLU, 80 mg/kg*** | ***4*** | ***3*** | ***7.474517*** | ***4.427945*** | ***0.001*** | ***0.011*** |
| ***Untreated*** | ***ATI-2307, 1 mg/kg + FLU, 80 mg/kg*** | ***9*** | ***3*** | ***15.81718*** | ***6.261723*** | ***2.77E-06*** | ***3.88E-05*** |
| ATI-2307, 1 mg/kg | ATI-2307, 2 mg/kg, 3 Doses | 5 | 5 | 0.04949 | 7.278558 | 0.962 | 1 |
| ATI-2307, 2 mg/kg | ATI-2307, 2 mg/kg, 3 Doses | 8 | 5 | -1.25531 | 10.88599 | 0.236 | 1 |
