# Supplementary material for: Pharmacodynamics of ATI-2307 in a rabbit model of cryptococcal meningoencephalitis
Source: Antimicrob Agents Chemother. 2023 Sep 20;67(10):e00818-23. doi: 10.1128/aac.00818-23 (PMC10583688; doi:10.1128/aac.00818-23)
Supplement: Supplemental Table 2 — Linear mixed effects model summary. [file aac.00818-23-s0003.docx]

| Supplemental Table 2: Linear Mixed Effects Model of the Fungal Burden in the CSF | | | |
| --- | --- | --- | --- |
|  | **Effects of Treatment Group & Day Post Infection on Fungal Burden** | | |
| *Predictors* | *Estimates* | *CI* | *p* |
| (Intercept) | 4.41 | 3.73 – 5.08 | **<0.001** |
| FLU, 80 mg/kg | 0.66 | -0.38 – 1.71 | 0.213 |
| Amphotericin B | 1.09 | 0.06 – 2.11 | **0.037** |
| ATI-2307, 1 mg/kg | 0.02 | -0.91 – 0.96 | 0.959 |
| ATI-2307, 2 mg/kg | 0.44 | -0.60 – 1.49 | 0.405 |
| ATI-2307, 3 mg/kg | 0.74 | -0.33 – 1.81 | 0.173 |
| ATI-2307, 1 mg/kg + FLU, 80 mg/kg | 0.86 | -0.20 – 1.92 | 0.113 |
| Day Post Infection | 0.10 | 0.02 – 0.18 | **0.012** |
| FLU, 80 mg/kg * Day Post Infection | -0.32 | -0.46 – -0.19 | **<0.001** |
| Amphotericin B * Day Post Infection | -0.42 | -0.53 – -0.31 | **<0.001** |
| ATI-2307, 1 mg/kg:Day Post Infection | -0.30 | -0.41 – -0.20 | **<0.001** |
| ATI-2307, 2 mg/kg:Day Post Infection | -0.53 | -0.66 – -0.39 | **<0.001** |
| ATI-2307, 3 mg/kg:Day Post Infection | -0.39 | -0.54 – -0.24 | **<0.001** |
| ATI-2307, 1 mg/kg + FLU, 80 mg/kg:ExpDay | -0.64 | -0.79 – -0.49 | **<0.001** |
| **Random Effects** | | | |
| σ^2^ | 0.51 | | |
| τ_00_ _RabbitID_ | 0.04 | | |
| ICC | 0.06 | | |
| N _RabbitID_ | 38 | | |
| Observations | 117 | | |
| Marginal R^2^ / Conditional R^2^ | 0.800 / 0.813 | | |
