# Supplementary material for: Pharmacodynamics of ATI-2307 in a rabbit model of cryptococcal meningoencephalitis
Source: Antimicrob Agents Chemother. 2023 Sep 20;67(10):e00818-23. doi: 10.1128/aac.00818-23 (PMC10583688; doi:10.1128/aac.00818-23)
Supplement: Supplemental Table 3 — Results of pairwise comparisons of estimated marginal means. [file aac.00818-23-s0004.docx]

| **Supplemental Table 3 – Pairwise Comparison of Estimated Marginal Means of Treatment Groups from Linear Mixed Effects Model** | | | | | |
| --- | --- | --- | --- | --- | --- |
| **Pairwise Comparison** | **Estimate** | **SE** | **DF** | **T Ratio** | **Bonferroni Adjusted P Value** |
| ***Untreated - (FLU, 80 mg/kg)*** | ***1.337215616*** | ***0.339265988*** | ***51.66621983*** | ***3.941496244*** | ***0.006842779*** |
| ***Untreated - Amphotericin B*** | ***1.526978216*** | ***0.365924175*** | ***51.37930037*** | ***4.172936143*** | ***0.003252509*** |
| ***Untreated - (ATI-2307, 1 mg/kg)*** | ***1.879949278*** | ***0.307395617*** | ***52.36054416*** | ***6.115732219*** | ***3.46E-06*** |
| ***Untreated - (ATI-2307, 2 mg/kg)*** | ***2.476138927*** | ***0.260587037*** | ***54.31504926*** | ***9.502156945*** | ***1.07E-11*** |
| ***Untreated - (ATI-2307, 3 mg/kg)*** | ***1.647437278*** | ***0.339532536*** | ***51.79404352*** | ***4.85207485*** | ***0.000325102*** |
| ***Untreated - (ATI-2307, 2 mg/kg, 3 Doses)*** | ***1.91623392*** | ***0.330544306*** | ***46.60476614*** | ***5.797207465*** | ***1.57E-05*** |
| ***Untreated - (COMBO(ATI-2307, 1 mg/kg + FLU, 80 mg/kg))*** | ***3.142192345*** | ***0.353288464*** | ***58.59147444*** | ***8.894126648*** | ***5.10E-11*** |
| (FLU, 80 mg/kg) - Amphotericin B | 0.1897626 | 0.414736693 | 51.55474784 | 0.457549581 | 1 |
| (FLU, 80 mg/kg) - (ATI-2307, 1 mg/kg) | 0.542733662 | 0.364140204 | 52.30752681 | 1.490452458 | 1 |
| ***(FLU, 80 mg/kg) - (ATI-2307, 2 mg/kg)*** | ***1.138923311*** | ***0.325594267*** | ***53.53125126*** | ***3.497983307*** | ***0.02665151*** |
| (FLU, 80 mg/kg) - (ATI-2307, 3 mg/kg) | 0.310221662 | 0.391648268 | 51.88882981 | 0.792092517 | 1 |
| (FLU, 80 mg/kg) - (ATI-2307, 2 mg/kg, 3 Doses) | 0.579018303 | 0.383882223 | 47.97012198 | 1.508322785 | 1 |
| ***(FLU, 80 mg/kg) - (COMBO(ATI-2307, 1 mg/kg + FLU, 80 mg/kg))*** | ***1.804976728*** | ***0.403631963*** | ***57.0040522*** | ***4.471837945*** | ***0.001049253*** |
| Amphotericin B - (ATI-2307, 1 mg/kg) | 0.352971061 | 0.389097905 | 51.96995995 | 0.907152306 | 1 |
| Amphotericin B - (ATI-2307, 2 mg/kg) | 0.94916071 | 0.353285604 | 52.92414644 | 2.686666818 | 0.269407092 |
| Amphotericin B - (ATI-2307, 3 mg/kg) | 0.120459062 | 0.414954765 | 51.6401408 | 0.290294442 | 1 |
| Amphotericin B - (ATI-2307, 2 mg/kg, 3 Doses) | 0.389255703 | 0.407632988 | 48.15815615 | 0.954917082 | 1 |
| ***Amphotericin B - (COMBO(ATI-2307, 1 mg/kg + FLU, 80 mg/kg))*** | ***1.615214128*** | ***0.426283771*** | ***56.18400579*** | ***3.789058461*** | ***0.010380573*** |
| (ATI-2307, 1 mg/kg) - (ATI-2307, 2 mg/kg) | 0.596189649 | 0.292236688 | 54.81210414 | 2.040091728 | 1 |
| (ATI-2307, 1 mg/kg) - (ATI-2307, 3 mg/kg) | -0.232512 | 0.364388557 | 52.41937682 | -0.638088093 | 1 |
| (ATI-2307, 1 mg/kg) - (ATI-2307, 2 mg/kg, 3 Doses) | 0.036284642 | 0.356028392 | 47.8622948 | 0.101915023 | 1 |
| ***(ATI-2307, 1 mg/kg) - (COMBO(ATI-2307, 1 mg/kg + FLU, 80 mg/kg))*** | ***1.262243067*** | ***0.377239203*** | ***58.36590901*** | ***3.346001836*** | ***0.040272102*** |
| (ATI-2307, 2 mg/kg) - (ATI-2307, 3 mg/kg) | -0.828701648 | 0.325871998 | 53.67352267 | -2.5430281 | 0.389368704 |
| (ATI-2307, 2 mg/kg) - (ATI-2307, 2 mg/kg, 3 Doses) | -0.559905007 | 0.316496058 | 47.87540463 | -1.76907419 | 1 |
| (ATI-2307, 2 mg/kg) - (COMBO(ATI-2307, 1 mg/kg + FLU, 80 mg/kg)) | 0.666053418 | 0.340180769 | 61.22284647 | 1.957939659 | 1 |
| (ATI-2307, 3 mg/kg) - (ATI-2307, 2 mg/kg, 3 Doses) | 0.268796641 | 0.384117812 | 48.06510277 | 0.69977656 | 1 |
| (ATI-2307, 3 mg/kg) - (COMBO(ATI-2307, 1 mg/kg + FLU, 80 mg/kg)) | 1.494755066 | 0.403856032 | 57.10009168 | 3.701207732 | 0.013571348 |
| (ATI-2307, 2 mg/kg, 3 Doses) - (COMBO(ATI-2307, 1 mg/kg + FLU, 80 mg/kg)) | 1.225958425 | 0.396329269 | 53.13158624 | 3.093282582 | 0.088282553 |
