# Supplementary material for: Pharmacodynamics of ATI-2307 in a rabbit model of cryptococcal meningoencephalitis
Source: Antimicrob Agents Chemother. 2023 Sep 20;67(10):e00818-23. doi: 10.1128/aac.00818-23 (PMC10583688; doi:10.1128/aac.00818-23)
Supplement: Supplemental Table 4 — Results of Pairwise Comparisons of EFAs. [file aac.00818-23-s0005.docx]

| **Supplemental Table 4 – Pairwise Comparisons of Linear Slopes (Effective Fungicidal Activity) for Treatment Groups** | | | | | |
| --- | --- | --- | --- | --- | --- |
| **Pairwise Comparison** | **Estimate** | **SE** | **DF** | **T Ratio** | **Bonferroni Adjusted P Value** |
| ***Untreated - (FLU, 80 mg/kg)*** | ***0.347532*** | ***0.073022*** | ***122.0332*** | ***4.759273*** | ***0.000151*** |
| ***Untreated - Amphotericin B*** | ***0.443774*** | ***0.068012*** | ***81.70661*** | ***6.524989*** | ***1.49E-07*** |
| ***Untreated - (ATI-2307, 1 mg/kg)*** | ***0.363651*** | ***0.063276*** | ***103.6857*** | ***5.747048*** | ***2.57E-06*** |
| ***Untreated - (ATI-2307, 2 mg/kg)*** | ***0.532235*** | ***0.059101*** | ***109.6292*** | ***9.005494*** | ***2.12E-13*** |
| ***Untreated - (ATI-2307, 3 mg/kg)*** | ***0.395689*** | ***0.077593*** | ***135.4964*** | ***5.099528*** | ***3.15E-05*** |
| ***Untreated - (ATI-2307, 2 mg/kg, 3 Doses)*** | ***0.45075*** | ***0.071286*** | ***115.604*** | ***6.323125*** | ***1.40E-07*** |
| ***Untreated - (COMBO(ATI-2307, 1 mg/kg + FLU, 80 mg/kg))*** | ***0.68301*** | ***0.079176*** | ***132.8273*** | ***8.626484*** | ***4.71E-13*** |
| (FLU, 80 mg/kg) - Amphotericin B | 0.096242 | 0.08083 | 97.57974 | 1.190679 | 1 |
| (FLU, 80 mg/kg) - (ATI-2307, 1 mg/kg) | 0.016118 | 0.076888 | 116.045 | 0.209636 | 1 |
| (FLU, 80 mg/kg) - (ATI-2307, 2 mg/kg) | 0.184703 | 0.07349 | 121.2856 | 2.513295 | 0.371627 |
| (FLU, 80 mg/kg) - (ATI-2307, 3 mg/kg) | 0.048157 | 0.089043 | 137.1362 | 0.540827 | 1 |
| (FLU, 80 mg/kg) - (ATI-2307, 2 mg/kg, 3 Doses) | 0.103218 | 0.083604 | 122.9922 | 1.234608 | 1 |
| ***(FLU, 80 mg/kg) - (COMBO(ATI-2307, 1 mg/kg + FLU, 80 mg/kg))*** | ***0.335477*** | ***0.090425*** | ***135.0693*** | ***3.710002*** | ***0.008451*** |
| Amphotericin B - (ATI-2307, 1 mg/kg) | -0.08012 | 0.072146 | 80.27356 | -1.11057 | 1 |
| Amphotericin B - (ATI-2307, 2 mg/kg) | 0.088461 | 0.068514 | 81.42881 | 1.291132 | 1 |
| Amphotericin B - (ATI-2307, 3 mg/kg) | -0.04809 | 0.084982 | 110.4114 | -0.56583 | 1 |
| Amphotericin B - (ATI-2307, 2 mg/kg, 3 Doses) | 0.006975 | 0.079265 | 92.12542 | 0.088002 | 1 |
| Amphotericin B - (COMBO(ATI-2307, 1 mg/kg + FLU, 80 mg/kg)) | 0.239235 | 0.086429 | 108.9595 | 2.767983 | 0.185632 |
| (ATI-2307, 1 mg/kg) - (ATI-2307, 2 mg/kg) | 0.168584 | 0.063816 | 102.9818 | 2.641733 | 0.266955 |
| (ATI-2307, 1 mg/kg) - (ATI-2307, 3 mg/kg) | 0.032038 | 0.081242 | 129.0293 | 0.394356 | 1 |
| (ATI-2307, 1 mg/kg) - (ATI-2307, 2 mg/kg, 3 Doses) | 0.087099 | 0.075241 | 110.098 | 1.157604 | 1 |
| ***(ATI-2307, 1 mg/kg) - (COMBO(ATI-2307, 1 mg/kg + FLU, 80 mg/kg))*** | ***0.319359*** | ***0.082755*** | ***126.7567*** | ***3.859105*** | ***0.005049*** |
| (ATI-2307, 2 mg/kg) - (ATI-2307, 3 mg/kg) | -0.13655 | 0.078034 | 134.7388 | -1.74983 | 1 |
| (ATI-2307, 2 mg/kg) - (ATI-2307, 2 mg/kg, 3 Doses) | -0.08149 | 0.071765 | 114.8941 | -1.13544 | 1 |
| (ATI-2307, 2 mg/kg) - (COMBO(ATI-2307, 1 mg/kg + FLU, 80 mg/kg)) | 0.150775 | 0.079608 | 132.1104 | 1.893966 | 1 |
| (ATI-2307, 3 mg/kg) - (ATI-2307, 2 mg/kg, 3 Doses) | 0.055061 | 0.087624 | 133.4769 | 0.628376 | 1 |
| (ATI-2307, 3 mg/kg) - (COMBO(ATI-2307, 1 mg/kg + FLU, 80 mg/kg)) | 0.287321 | 0.094155 | 142.8196 | 3.051569 | 0.076021 |
| (ATI-2307, 2 mg/kg, 3 Doses) - (COMBO(ATI-2307, 1 mg/kg + FLU, 80 mg/kg)) | 0.23226 | 0.089029 | 131.4129 | 2.608811 | 0.283875 |
