# Supplementary material for: Pharmacodynamics of ATI-2307 in a rabbit model of cryptococcal meningoencephalitis
Source: Antimicrob Agents Chemother. 2023 Sep 20;67(10):e00818-23. doi: 10.1128/aac.00818-23 (PMC10583688; doi:10.1128/aac.00818-23)
Supplement: Supplemental Table 5 — Brain Tissue Fungal Burden and Summary Statistics. [file aac.00818-23-s0006.docx]

| **Supplemental Table 5 – Fungal Burden in Brain Tissue** | | | | | | | | |
| --- | --- | --- | --- | --- | --- | --- | --- | --- |
| **Group** | **Tissue** | **Mean CFU/g** | **SD** | **N** | **Mean Day Post Infection** | **SEM** | **95% CI Upper Limit** | **95% CI Lower Limit** |
| Untreated | Brainstem/Cerebellum | 6.104873 | 0.434287 | 10 | 11.1 | 0.137334 | 6.415543 | 5.794203 |
| Untreated | Cerebrum | 6.464354 | 0.372516 | 10 | 11.1 | 0.1178 | 6.730836 | 6.197873 |
| Untreated | Vitreous Humor | 2.739863 | 1.049023 | 10 | 11.1 | 0.33173 | 3.490289 | 1.989437 |
| FLU, 80 mg/kg | Brainstem/Cerebellum | 4.756002 | 0.551583 | 5 | 10 | 0.246675 | 5.440883 | 4.071121 |
| FLU, 80 mg/kg | Cerebrum | 4.427128 | 0.415376 | 5 | 10 | 0.185762 | 4.942885 | 3.91137 |
| FLU, 80 mg/kg | Vitreous Humor | 0 | 0 | 5 | 10 | 0 | 0 | 0 |
| Amphotericin B | Brainstem/Cerebellum | 2.634497 | 0.47187 | 4 | 14 | 0.235935 | 3.385347 | 1.883647 |
| Amphotericin B | Cerebrum | 2.772498 | 0.606328 | 4 | 14 | 0.303164 | 3.737301 | 1.807695 |
| Amphotericin B | Vitreous Humor | 0 | 0 | 4 | 14 | 0 | 0 | 0 |
| ATI-2307, 1 mg/kg | Brainstem/Cerebellum | 4.894754 | 0.685625 | 7 | 11.28571 | 0.259142 | 5.528851 | 4.260657 |
| ATI-2307, 1 mg/kg | Cerebrum | 5.464117 | 0.483288 | 7 | 11.28571 | 0.182666 | 5.911084 | 5.017151 |
| ATI-2307, 1 mg/kg | Vitreous Humor | 0 | 0 | 7 | 11.28571 | 0 | 0 | 0 |
| ATI-2307, 2 mg/kg | Brainstem/Cerebellum | 4.146184 | 0.691361 | 9 | 9.888889 | 0.230454 | 4.677611 | 3.614757 |
| ATI-2307, 2 mg/kg | Cerebrum | 4.678866 | 1.058063 | 9 | 9.888889 | 0.352688 | 5.492166 | 3.865567 |
| ATI-2307, 2 mg/kg | Vitreous Humor | 0 | 0 | 9 | 9.888889 | 0 | 0 | 0 |
| ATI-2307, 3 mg/kg | Brainstem/Cerebellum | 5.068614 | 0.036038 | 2 | 10 | 0.025483 | 5.3924 | 4.744827 |
| ATI-2307, 3 mg/kg | Cerebrum | 5.474469 | 0.045451 | 2 | 10 | 0.032139 | 5.882828 | 5.066109 |
| ATI-2307, 3 mg/kg | Vitreous Humor | NA | NA | 2 | 10 | NA | NA | NA |
| ATI-2307, 2 mg/kg, 3 Doses | Brainstem/Cerebellum | 5.075213 | 0.718515 | 6 | 10 | 0.293332 | 5.829248 | 4.321177 |
| ATI-2307, 2 mg/kg, 3 Doses | Cerebrum | 5.159521 | 0.412029 | 6 | 10 | 0.16821 | 5.59192 | 4.727123 |
| ATI-2307, 2 mg/kg, 3 Doses | Vitreous Humor | 0 | 0 | 5 | 10 | 0 | 0 | 0 |
| ATI-2307, 1 mg/kg + FLU, 80 mg/kg | Brainstem/Cerebellum | 3.257344 | 0.600888 | 5 | 8.8 | 0.268725 | 4.003445 | 2.511243 |
| ATI-2307, 1 mg/kg + FLU, 80 mg/kg | Cerebrum | 3.273094 | 0.635983 | 5 | 8.8 | 0.28442 | 4.062771 | 2.483417 |
| ATI-2307, 1 mg/kg + FLU, 80 mg/kg | Vitreous Humor | 0 | 0 | 5 | 8.8 | 0 | 0 | 0 |
