# Supplementary material for: Pharmacodynamics of ATI-2307 in a rabbit model of cryptococcal meningoencephalitis
Source: Antimicrob Agents Chemother. 2023 Sep 20;67(10):e00818-23. doi: 10.1128/aac.00818-23 (PMC10583688; doi:10.1128/aac.00818-23)
Supplement: Supplemental Table 6 — Brain Tissue Concentrations and Collection Times. [file aac.00818-23-s0007.docx]

| **Supplemental Table 6 – Concentrations of ATI-2307 in Terminal Brain Tissue and Serial CSF Samples, and Collection Times for Tissue** | | | | | | |
| --- | --- | --- | --- | --- | --- | --- |
| **Treatment Group** | **CSF**  **Day 7,**  **ng/mL** | **CSF**  **Day 10,**  **ng/mL** | **Cerebrum**  **mg/L** | **Cerebellum/Brainstem**  **mg/L** | **Meninges**  **mg/L** | **Mean Brain Collection Time (hour post infection)** |
| ATI-2307, 1 mg/kg | 9.82  (3.88)  N = 8 | 8.80  (2.71)  N = 6 | 0.59 (0.08) N = 6 | 0.69 (0.13) N = 6 | Not Collected | 273 |
| ATI-2307, 2 mg/kg | 15.3  (15.2)  N = 8 | 24.8  (8.23)  N = 8 | 0.9 (0.17) N = 10 | 1.03 (0.25) N = 10 | 4.35  (0.23)  N = 4 | 237 |
| ATI-2307, 2 mg/kg, 3 Doses | 0.444  (0.267)  N = 6 | 0.511  (0.573)  N = 6 | 0.26 (0.06) N = 6 | 0.26 (0.07) N = 6 | 0.66  (0.34)  N = 5 | 238 |
| ATI-2307, 3 mg/kg | 27.3  (10.9)  N = 5 | 422  (590)  N = 2 | 1.14 (0.32) N = 2 | 1.33 (0.35) N = 2 | Not Collected | 240 |
| ATI-2307, 1 mg/kg + FLU, 80 mg/kg | 7.4  (2.79)  N = 4 | 11.2  (0.635)  N = 3 | 0.43 (0.13) N = 5 | 0.44 (0.13) N = 5 | Not Collected | 212 |
| **Data are pooled from three experiments*  *Data are the mean values with (SD)* | | | | | | |
